# Synthesis and screening of antibacterial and antifungal activity of 5-chloro-1,3-benzoxazol-2(3 h)-one derivatives

**DOI:** 10.1186/2191-2858-2-29

**Published:** 2012-08-01

**Authors:** Priya R Modiya, Chhaganbhai N Patel

**Affiliations:** 1 Department of Pharmaceutical Chemistry, Shri Sarvajanik Pharmacy College, Gujarat Technological University, Arvind Baug, Mehsana, 384001, Gujarat, India

**Keywords:** Antibacterial, Antifungal, Benzoxazolinone, *p*-aminobenzoic acid, Sulfanilamide, Triazole

## Abstract

**Background:**

An antibacterial is a substance that either kills bacteria or slows their growth. Antifungal are the agents that use drugs for treatment of fungal infections. 5-Chloro-1,3-benzoxazol-2(3 H)-one (5-Chloro Benzoxazolinone) contains an azole ring structure. Numbers of azole compounds are reported as antibacterial and antifungal agents. Benzoxazolinones naturally occur in plants. They play a role as defense compounds against bacteria, fungi, and insects.

**Results:**

In this article, synthesis of six Benzoxazolinone derivatives with various substituents is presented. Benzoxazolinone substituted with *p*-aminobenzoic acids and sulphanilamide derivatives. The above both substituents are reported as potent antimicrobial agents. Attachment with azole leads to increase its potency. The other substituents are 2,4-dichlorobezylchloride. The same rings are found in miconazole and this may lead to increase its antifungal activity. Fluconazole also contains triazole moiety and triazole is having other numbers of activity like antimicrobial, anti-inflammatory, local anesthetic, antiviral, anticancer, antimalarial, etc. Here, there is a substitution for azole ring at 5-Chloro position which might increase antibacterial and antifungal activity. The synthesis and interpretation of six final compounds and three intermediates are presented in this article. Synthesis of 5-Chloro Benzoxazolinone derivatives substituted with Halogenated rings, sulfonated and benzylated derivatives and azole derivatives. There is a synthesis of P2A, P2B, P4A, P4B, P5A, and P6A compounds and their structures were characterized by UV–Visible, IR, MASS spectroscopy, and NMR spectroscopy.

**Conclusions:**

The antibacterial activity of all six compounds is measured against various Gram-positive and Gram-negative bacteria and against fungi. Compounds P4A and P4B have good antibacterial and antifungal activity, half of the Ampicillin and Cephalexin. P4A, P4B, P6A have good activity against *Staphylococcus aureus* and *Escherichia coli.* Compound P2B has good antifungal activity, half of the Miconazole against *Candida albicans*. P2A, P2B, P5A, P6A have almost equal antibacterial activity.

## Background

An antibacterial is a substance that either kills bacteria or slows their growth. An antifungal drug is a medication used to treat fungal infection such as athlete’s foot, ring worm, candidiasis (thrust), serious systemic infections such as *cryptococcal meningitis* and other. The benzoxazolinone ring is having number of activities. Benzoxazolinone derivatives naturally occur in plants. It has natural defense mechanism in plants against bacteria, fungi, and insects. Antibiotics Qustinamycin and *N*-acetyl Quistinamycin belong to this group. As described in literature review, it has potential as antibacterial and antifungal agents. A number of azoles are used in fungi and bacterial infection. So, here we are going to synthesize various derivatives of benzoxazolinone with various substituents.

## Methods

**Figure 1:**
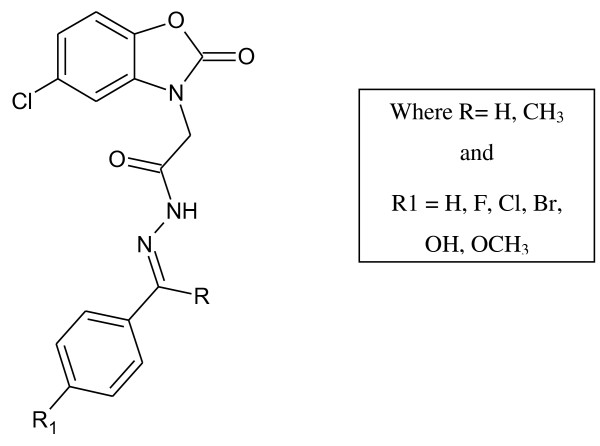
**5-Chloro-2(3 H)-benzoxazolinone-3-acetyl-2-(*p*-substituted benzalhydrazone and 5-chloro-2(3 H)-benzoxazolinone-3-acetyl-2-(*p*-substituted acetophenone) hydrazone derivatives.**

**Figure 2:**
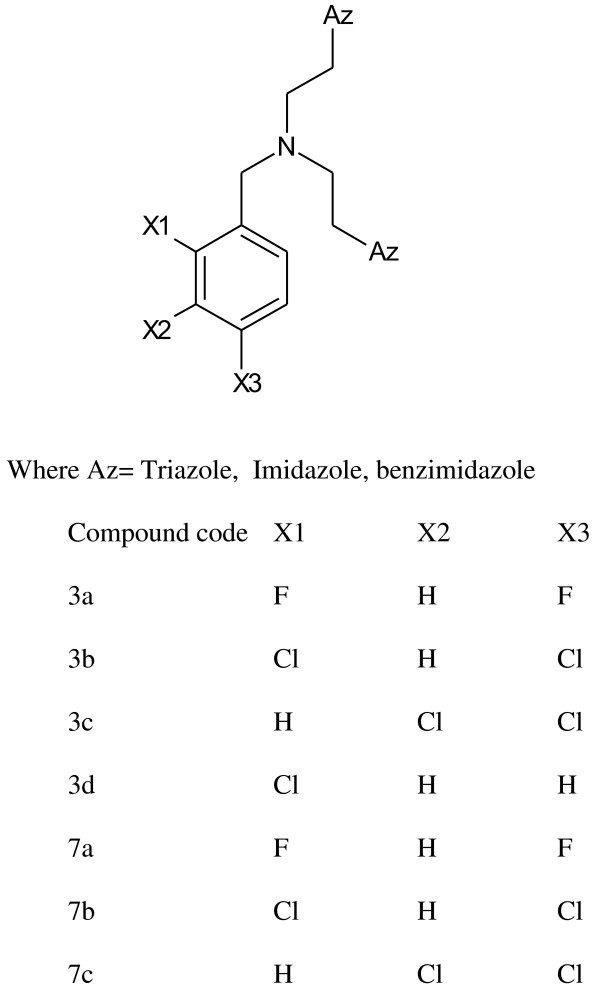
***N*-(2-(1 H-1,2,4-triazol-1-yl)ethyl)-*N*-(2,4-difluorobenzyl)-2-(1 H-1,2,4-triazol-1-yl)ethanamine hydrochloride, *N*-(2-(1 H-1,2,4-triazol-1-yl)ethyl)-*N*-(2,4-dichlorobenzyl)-2-(1 H-1,2,4-triazol-1-yl)ethanamine hydrochloride, *N*-(2-(1 H-1,2,4-triazol-1-yl)ethyl)-*N*-(3,4-dichlorobenzyl)-2-(1 H-1,2,4-triazol-1-yl)ethanamine hydrochloride.**

**Figure 3:**
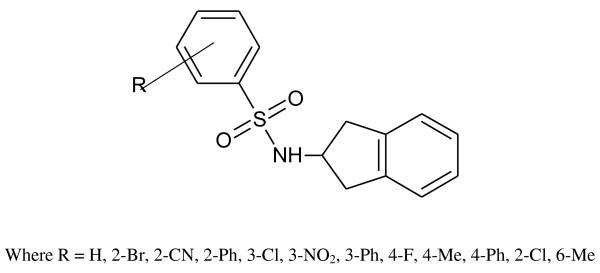
Benzoxazole benzenesulfonamide derivatives.

**Figure 4:**
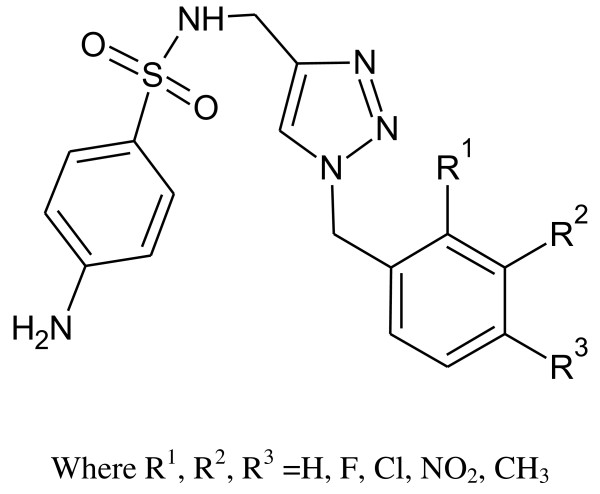
**4-Amino-*N*-((1-pentyl-1 H-1,2,3-triazol-4-yl)methyl)benzenesulfonamide, 4-amino-*N*-((1-hexyl-1 H-1,2,3-triazol-4-yl)methyl)benzenesulfonamide.**

5-Chloro-2(3 H)-benzoxazolinone-3-acetyl-2-(*p*-substituted benzalhydrazone) and 5-chloro-2(3 H)-benzoxazolinone-3-acetyl-2-(*p*-substituted acetophenone) hydrazone derivatives were synthesized (Figure 1). Among them, the analytical data of five original compounds were given. In this study, the microwave synthesis method and antimicrobial evaluation of all the compounds were also reported for the first time. The minimum inhibition concentration (MIC) values of the compounds were determined by the Microdilution method using two Gram-positive bacteria (*Staphylococcus aureus, Bacillus subtilis*), two Gram-negative bacteria (*Pseudomonas aeruginosa, Escherichia coli*), and two yeast-like fungi (*Candida albicans, Candida parapsilosis*) [1]. Fang et al. [2] have reported *N*-(2-(1 H-1,2,4-triazol-1-yl)ethyl)-*N*-(2,4-difluorobenzyl)-2-(1 H-1,2,4-triazol-1-yl)ethanamine hydrochloride, *N*-(2-(1 H-1,2,4-triazol-1-yl)ethyl)-*N*-(2,4-dichlorobenzyl)-2-(1 H-1,2,4-triazol-1-yl)ethanamine hydrochloride, *N*-(2-(1 H-1,2,4-triazol-1-yl)ethyl)-*N*-(3,4-dichlorobenzyl)-2-(1 H-1,2,4-triazol-1-yl)ethanamine hydrochloride and number of other compounds having potent antibacterial and antifungal activity (Figure 2). Miconazole contains two 2,4-dichlorobenzyl ring and imidazole as azole. As per the SAR study of the above compounds, the halogenated ring is responsible for the antifungal activity potential and azoles are also present. So, we will attach the halogenated ring to benzoxazolinone and synthesize the P2A and P2B compounds. A series of novel benzoxazole benzenesulfonamides was synthesized (Figure 3) as inhibitors of fructose-1,6-bisphosphatase (FBPase-1), and they are proved as antibacterial agents [3]. The sulfanilamide derivatives and *p*-aminobenzoic acid are very good antimicrobial agents as per traditional drug review. So, here we will attach the sulfanilamide and *p*-aminobenzoic acid with benzoxazolinone. Triazole derivatives are having number of activities. Moreover, fluconazole is having two triazole ring and 2,4-difluorobenzyl ring. Wang et al. [4] have reported triazole substituted with sulfanilamide as potent antimicrobial agent (Figure 4) compounds such as 4-amino-*N*-((1-pentyl-1 H-1,2,3-triazol-4-yl)methyl)benzenesulfonamide, 4-amino-*N*-((1-hexyl-1 H-1,2,3-triazol-4-yl)methyl)benzenesulfonamide and other compounds [4].

## Results and discussion

### Antibacterial screening

The microbiological assay is based upon a comparison of inhibition of growth of micro-organisms by measured concentrations of test compounds with that produced by known concentration of a standard antibiotic. Two methods generally employed are *turbidometric (tube-dilution) method* and *cylinder plate (cup-plate) method*. In the turbidometric method, inhibition of growth of microbial culture in a uniform solution of antibiotic in a fluid medium is measured. It is compared with the synthesized compounds. Here, the presence or absence of growth is measured. The cylinder plate method depends upon diffusion of antibiotic from a vertical cylinder through a solidified agar layer in a Petridish or plate to an extent such that growth of added micro-organisms is prevented entirely in a zone around the cylinder containing solution of the antibiotics. The cup-plate method is simple and measurement of inhibition of microorganisms is also easy. Here, we have use this method for antibacterial screening of the test compounds. The media was prepared from nutrient agar 2%, peptone 1%, beef extract 1%, sodium chloride 0.5%, and distilled water up to 100 mL. All the ingredients were weighed and added to water. This solution was heated on water bath for about one and half-hour till it became clear. This nutrient media was sterilized by autoclave. The antibacterial and antifungal activity was measured against *Bacillus subtillis* (MTCC-212), *Staphylococcus aureus* (MTCC-737) were used as Gram-positive bacteria, *Escherichia coli* (NCLM-2066) were used as Gram-negative bacteria and *Candida albicans* (MTCC-227) was used as fungi for this study. The master culture was prepared on agar slant of the above nutrient media and kept in refrigerator. The working culture was prepared from it by weekly transferred in nutrient agar medium [5,6].

#### Preparation of inoculums

In the aseptic condition from the working culture, small amount of culture was transferred to about 10–15 mL of sterile normal saline (0.9% NaCl solution). This solution was gently mixed and used for the antibacterial activity. About 0.5 mL of inoculum was added to the sterilized Petridish and melted agar cooled to 45°C was added, mixed gently, and allowed to solidify. Then, Watmann filter paper disk was kept in each plate which was soaked in test drug solutions. The solution was allowed to diffuse for a period of 90 min. The Petri dishes were then incubated at 37°C for 24 h after which zone of inhibition was measured.

#### Preparation of test solution

**Figure 5:**
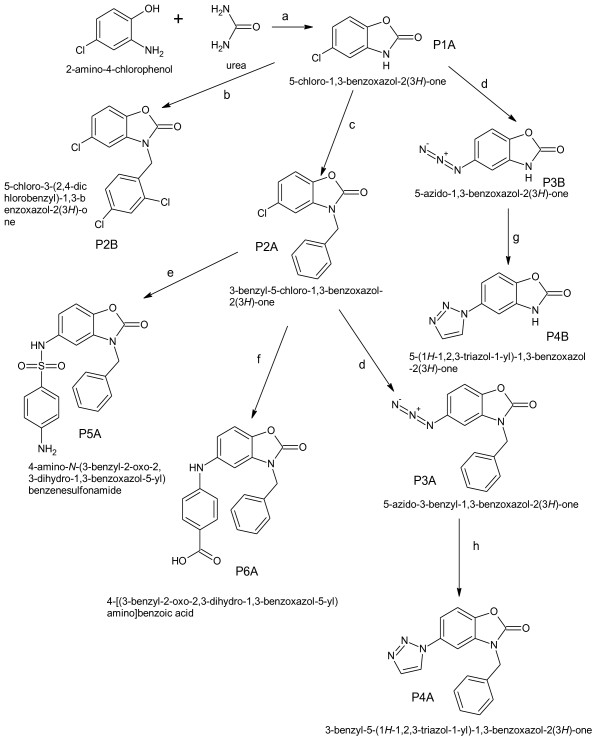
**Zone of inhibition of Ampicillin in *B. subtilis.***

**Figure 6:**
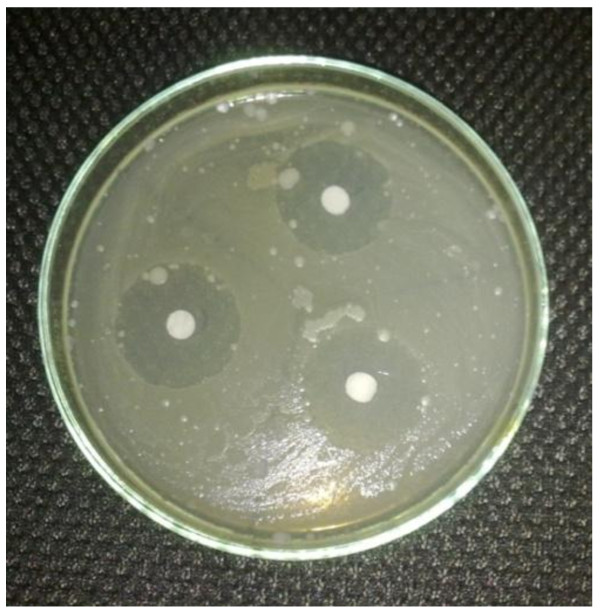
**Zone of inhibition of Cephalexin in *E. coli.***

**Figure 7:**
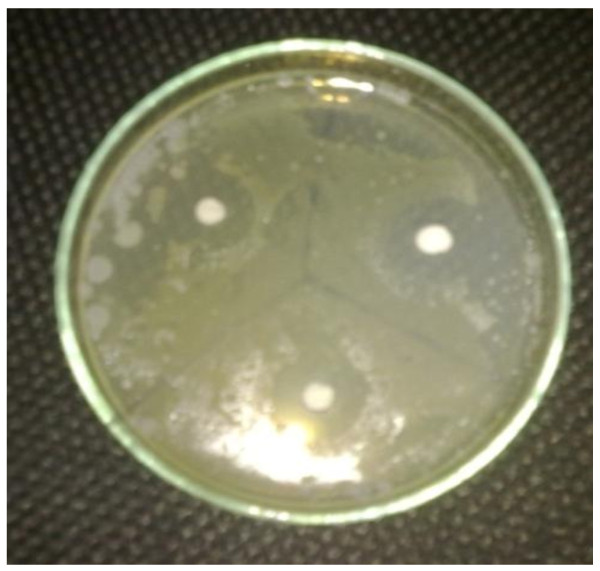
**Zone of inhibition of P4B in *S. ureu.***

**Figure 8:**
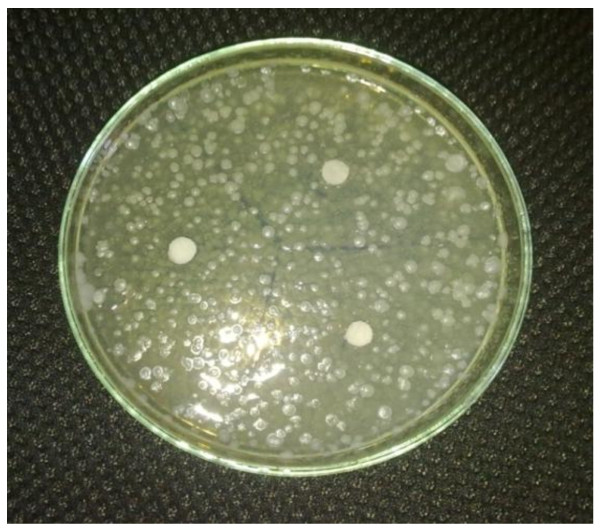
**Zone of inhibition of P2B in *C. albicans.***

**Figure 9:**
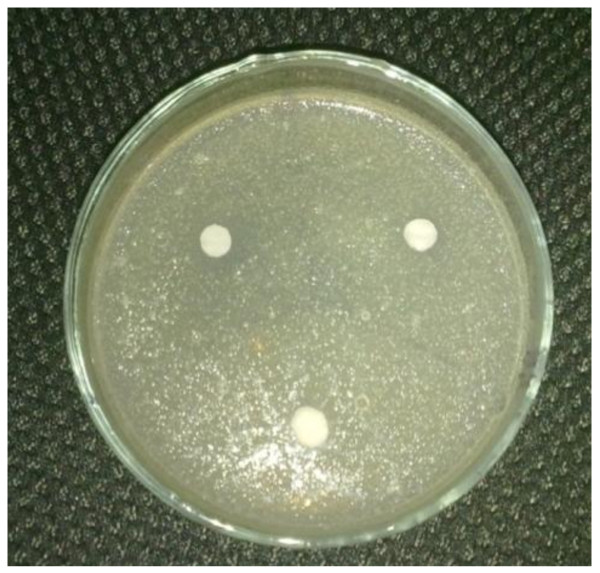
Histogram of antibacterial/antifungal act.

Specified quantity of the compound was weighed and dissolved in 5 mL of DMSO and further dilution was made to get the concentration of 500, 1000, and 1500 μg/mL. Similarly, the standard drugs Ampicillin, Cephalexin, and Miconazole were dissolved in appropriate quantity of water to obtain the concentration of 500, 1000, and 1500 μg/mL each. The images of zone of inhibition are given in Figures 5, 6, 7, and 8, and the results are shown in Table 1, and the histogram of antibacterial and antifungal activity is given in Figure 9.

**Table 1:** Zone of inhibition antibacterial activity of synthesized compounds

**Compound code/name**	**Concentration (mg/ml)**	**Zone of inhibition (mm)**			
		***B. subtilis***	***S. aureus***	***E. coli***	***C. albicans***
**P2A**	0.5	2	1	-	2
	1.0	4	3	2	4
	1.5	6	5	4	7
**P2B**	0.5	–	–	–	–
	1.0	1	2	3	3
	1.5	3	3	5	6
**P4A**	0.5	–	5	–	–
	1.0	6	7	2	3
	1.5	7	8	3	5
**P4B**	0.5	–	3	1	3
	1.0	4	5	3	4
	1.5	7	6	4	7
**P5A**	0.5	1	–	–	2
	1.0	5	3	–	5
	1.5	6	5	3	7
**P6A**	0.5	4	3	–	–
	1.0	6	5	2	4
	1.5	7	7	5	6
**Ampicillin**	0.5	10	11	8	–
	1.0	12	12	10	–
	1.5	14	14	12	–
**Cephalexin**	0.5	7	7	5	–
	1.0	9	8	8	–
	1.5	11	10	9	–
**Miconazole**	0.5	–	–	–	6
	1.0	–	–	–	7
	1.5	–	–	–	9

#### MIC

MIC is the lowest concentration of an antimicrobial that will inhibit the visible growth of a microorganism after overnight incubation. MIC values can be determined by a number of standard test procedures. The most commonly employed methods are the tube dilution and agar dilution methods. Serial dilutions are made of the products in bacterial growth media. The test organisms are then added to the dilutions of the products, incubated, and scored for growth. This procedure is a standard assay for antimicrobials. Minimum inhibitory concentrations are important in diagnostic laboratories to confirm resistance of microorganisms to an antimicrobial agent and also to monitor the activity of new antimicrobial agents. A MIC is generally regarded as the most basic laboratory measurement of the activity of an antimicrobial agent against an organism. The data derived from the test can be corrected with the knowledge of expected or measured compound level *in vivo* to predict the efficacy of compound. In this study, MIC was determined using “Serial tube dilution technique”. In this technique, the tubes of broth medium, containing graded doses of compounds, are inoculated with the test organisms. After suitable incubation, growth will occur in those tubes where the concentration of compound is below the inhibitory level and the culture will become turbid (cloudy). Therefore, growth will not occur above the inhibitory level and the tube will remain clear. Results are shown in Table 2[7].

**Table 2:** MIC of synthesized compounds

**Compound code/name**	**Concentration (μg/mL)**			
	***B. subtilis***	***S. aureus***	***E. coli***	***C. albicans***
**P2A**	>312	>312	>312	>312
**P2B**	>312	>312	>312	>156
**P4A**	>156	>156	>156	>156
**P4B**	>156	>156	>156	>156
**P5A**	>312	>312	>312	>156
**P6A**	>312	>156	>312	>312
**Ampicillin**	>39	>78.1	>1250	–
**Cephalexin**	>39	>78.1	>78.1	–
**Miconazole**	–	–	–	>78.1

#### Procedure

● Twelve test tubes were taken, nine of which were marked 1, 2, 3, 4, 5, 6, 7, 8, 9, and the rest three were assigned as *T*_M_ (medium), *T*_MC_ (medium + compound), and *T*_MI_ (medium + inoculum).

● 2 mL of nutrient broth medium was poured to each of the 12 test tubes.

● These test tubes were cotton plugged and sterilized in an autoclave for 15 lbs/sq. inch pressure.

● After cool 2 mL of the sample solution (5 mg/mL) was added to the first test tube and mixed well and then 2 mL of this content was transferred to the second test tube.

● The content of the second test tube was mixed well and again 2 mL of this mixture was transferred to the third test tube. This process of serial dilution was continued up to the ninth test tube.

● *l* of properly diluted inoculum was added to each of nine test tubes and mixed well.

● To the control test tube *T*_MC_, 2 mL of the sample was added, mixed well and 2 mL of this mixed content was discarded to check the clarity of the medium in presence of diluted solution of the compound.

● 10 μL of the inoculum was added to the control test tube *T*_MI_, observed the growth of the organism in the medium used.

● The control test tube *T*_M_, containing medium only was used to confirm the sterility of the medium.

● All the test tubes were incubated at 37°C for 18 h.

● The resultant concentration in all the nine test tubes will be 2.5 mg/mL, 1.25 mg/mL, 625, 312.5, 156.25, 78.1, 39.06, 19.53, 9.76 μg/mL.

## Experimental

**Scheme 1:**
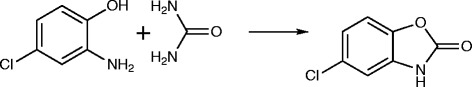
**Synthesis of 5-chloro-1,3-benzoxazol-2(3** ***H*)-one (P1A).**

All the chemicals used for the synthesis of title compounds were procured from Himedia Laboratories Pvt. Ltd., Mumbai, S. D. Fine Chem. Ltd., Mumbai, Finar Chemicals Ltd., Ahmedabad, Loba chemie Pvt. Ltd., Mumbai, Chemdyes Corporation, Ahmedabad, Spectrochem Pvt. Ltd., Mumbai. The scheme of synthesis is given in Scheme 1. The chemicals were used without further purification. All the melting points were determined in open capillaries and are uncorrected. Thin layer chromatography was performed on microscopic slides (2 × 7.5 cm^2^) coated with silica-Gel-G and spots were visualized under UV light. UV spectra were recorded in U.V-1700 Shimadzu spectrophotometer. IR spectra of all the compounds were recorded in KBr on FT-IR 8400 S Shimadzu spectrophotometer using KBr. Mass spectra were obtained using 2010EV LCMS Shimadzu instrument. The ^1^ H NMR was recorded on Bruker advanced–II NMR-400 MHz instruments using CDCl3/DMSO-d6 as solvent and tetramethylsilane as internal standard, chemical shifts were expressed as δvalues (ppm).

### Preparation of 5-chloro-1,3-benzoxazol-2(3 *H*)-one (P1A)

**Scheme 2:**
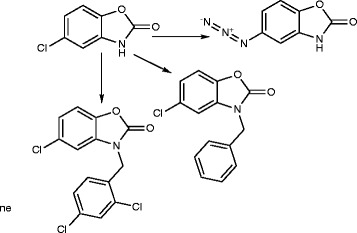
**Synthesis of 3-benzyl-5-chloro-1,3-benzoxazol-2(3** ***H*)-one (P2A), 5-chloro-3-(2,4-dichlorobenzyl)-1,3-benzoxazol-2(3 H)-one (P2B), 5-azido-1,3-benzoxazol-2(3** ***H*)-one (P3B).**

(7.15 g, 0.05 mol) 2-amino-4 chlorophenol was dissolved in 10 mL of Dimethyl formamide. (3 g, 0.05 mol) of urea was added in this mixture. The whole mixture was reflux for 3 h at 60°C till all the ammonia gas is liberated. Then, pour this mixture in ice-cold water with constant stirring and collect the precipitates. The product was recrystallized from Rectified ethanol. The compound was characterized by TLC, UV, IR, and melting point determination (Tables 3 and 4). Mobile phase of TLC was hexane:ethylacetate (3:2) [1] Scheme 2.

**Table 3:** Physical data of compounds

**Compound code**	**Molecular formula**	**Molecular weight (g/mol)**	**Melting point (°C)**	**Yield (%w/w)**	***R***_**f**_**value**
**P1A**	C_7_H_4_ClNO_2_	169.565	158	70.1	0.73
**P2A**	C_14_H_10_ClNO_2_	259.68	169	78.4	0.84
**P2B**	C_14_H_8_Cl_3_NO_2_	328.577	176	56.9	0.68
**P3A**	C_14_H_10_N_4_O_2_	266.25	176	70.17	0.78
**P3B**	C_7_H_4_N_4_O_2_	176.13	168–172	94.23	0.74
**P4A**	C_16_H_12_N_4_O_2_	292.29	135	37.03	0.87
**P4B**	C_9_H_6_N_4_O_2_	202.169	179–183	42.15	0.54
**P5A**	C_20_H_17_N_3_O_4_S	395.43	195–200	65.79	0.71
**P6A**	C_21_H_16_N_2_O_4_	360.36	182	54.02	0.72

**Table 4:** Spectral data of compounds

**Compound code**	**UV λ-max (cm)**	**IR KBr (cm**^**-1**^**)**	**Mass m/z (abundance)**	**H**^**1**^**NMR (CDCl**_**3**_**) signals, δ, multiplicity,*J*value (Hz)**
**P1A**	283.0	3055.03 (−NH–CO–),	–	–
		800–400(Ar–Cl),		
		1481.23(Cyclic Ester),		
		1600–1400 (C = C)		
		1782.10 (C = O)		
**P2A**	282.0	3051.18 (=N–CO–),	259(M^–^),	7.1 (s, 5H, Ar–H),
		800–400(Ar–Cl),	168(91),	4.2 (s, 2H, –CH_2_–),
		1481.23(Cyclic Ester),	223(35),	7.1, 7.0 (m, 2 H, –^3^CH–
		1600–1400 (C = C)	186(73),	^4^CH–),
		713.0 & 694.33 (Mono Substituted benzene ring)	113(164)	7.3, 7.2 (m, 1 H, –^6^CH)
		1774.39 (C = O)		
**P2B**	282.5	3055.03 (−NH–CO–),	326(M^–^),	–
		800–400(Ar–Cl),	168(158),	
		1620.09–1778.25 (1,2,4-trisubstituted benzene ring),	292.9(35),	
		1477.37(Cyclic Ester),	258.9(70),	
		1600–1450 (C = C),	218(105),	
		3050–3010 (−C–H str.),	181(146),	
		1778.25 (C = O)	159(167)	
**P3A**	282.0	3039.09 (=N–CO–),	–	–
		713.1 & 698.18 (Mono substituted benzene ring),		
		1481.23(Cyclic Ester),		
		1600–1400 (C = C),		
		3050–3010 (−C–H str),		
		1782.10 (C = O)		
**P3B**	282.0	3055.03 (−NH–CO–),	–	–
		1485.09(Cyclic Ester),		
		1600–1450 (C = C),		
		3050–3010 (−C–H str),		
		1782.10 (C = O)		
**P4A**	282.0	3058.89 (=N–CO–),	292(M^–^),	7.02 (s, 5 H, Ar–H),
		1485.09(Cyclic Ester),	168(106),	4.02 (s, 2 H, –CH_2_–),
		1600–1450 (C = C),	224(68),	7.8 (s, 1 H, ^6^CH),
		713.61 & 690.47 (Mono substituted benzene ring),	201(91),	7.36, 7.35 (m, 2 H, –^3^CH–^4^CH–),
		3050–3010 (−C–H str),	133(159)	7.1, 7.0 (d, 2 H, –CH = CH– in triazole)
		1762.82 (C = O)		
**P4B**	282.0	3055.03 (−NH–CO–),	202(M^–^),	
		1481.23(Cyclic Ester),	168(34),	
		1600–1400 (C = C),	134(68)	
		3050–3010 (−C–H str),		
		1782.10 (C = O)		
**P5A**	281.0 & 218.0	1261.36 & 1149.50 (−SO_2_NH–),	395(M^-^),	–
		3055.03 (=N–CO–),	167.9(227),	
		1620.09 (−NH_2_),	224(171)	
		1299.93 & 1149.50 (−S = O),	304(91)	
		1477.37(Cyclic Ester),	133(262)	
		1782.10 (C = O)		
**P6A**	282.0 & 219.0	1782.10 (−COOH)	360(M^–^),	7.02 (s, 5 H, Ar–H),
		3058.89 (=N–CO–),	310.8(49),	7.01,7.00 (d, 4 H, CH in PABA),
		713.60 & 694.33 (Mono substituted benzene ring),	168(192),	5.01 (s, 1 H, NH),
		1481.23(Cyclic Ester),	223.9(136),	11.51 (s, 1 H, –COOH),
		1600–1400 (C = C)	132(227)	4.3 (s, 2 H, –CH_2_–),
		1616.24 (C = O)		7.24, 7.22 (m, 1 H, ^6^CH),
				7.76,7.74 (m, 2 H, –^3^CH–^4^CH–)

### Preparation of 3-benzyl-5-chloro-1,3-benzoxazol-2(3 *H*)-one (P2A)

**Scheme 3:**
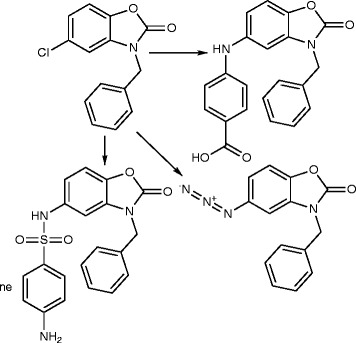
**Synthesis of 5-azido-3-benzyl-1,3-benzoxazol-2(3*H*)-one(P3A), 4-amino-*N*-(3-benzyl-2-oxo-2,3-dihydro-1,3-benzoxazol-5-yl)benzenesulfonamide (P5A), 4-[(3-benzyl-2-oxo-2,3-dihydro-1,3-benzoxazol-5-yl)amino]benzoic acid (P6A).**

(1.69 g, 0.010 mol) of P1A was added in 5 mL of acetonitrile in a round bottom flask. To this mixture, (1.26 g, 0.01 mol) of benzyl chloride (density 1.100 g/cm^3^) was added. The whole solution was reflux for 3 h at 60°C. Then, this solution was added in cold water with constant stirring. Product was collected and recrystallized from rectified ethanol. The compounds were characterized by TLC, UV, IR, MASS, and melting point determination (Tables 3 and 4). Mobile phase of TLC was hexane:ethyl acetate (3:2) [2] Scheme 3.

### Preparation of 5-chloro-3-(2,4-dichlorobenzyl)-1,3-benzoxazol-2(3Â H)-one (P2B)

(1.69 g, 0.01 mol) of P1A was added to the 5 ml of acetonitrile in a round bottom flask. To this mixture, (1.365 g, 0.01 mol) of 2,4-dichlorobenzyl chloride (density 1.386 g/cm^3^) was added. The whole mixture was refluxed for 4 h at 60°C and reaction was monitored by TLC. Then, resultant mixture was added in cold water with constant stirring. The product was collected and recrystallized from rectified ethanol. The compound was characterized by TLC, UV, IR, MASS, NMR, and melting point determination (Tables 3 and 4). Mobile phase of TLC was hexane:ethyl acetate (3:2) [2].

### Preparation of 5-azido-3-benzyl-1,3-benzoxazol-2(3 *H*)-one(P3A)

**Scheme 4:**
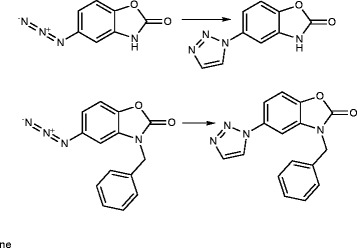
**Synthesis of 5-(1** ***H*-1,2,3-triazol-1-yl)-1,3-benzoxazol-2(3** ***H*)-one (P4B), 3-benzyl-5-(1*H*-1,2,3-triazol-1-yl)-1,3-benzoxazol-2(3*H*)-one (P4A).**

(2.59 g, 0.01 mol) of P2A was dissolved in 5 mL of dimethyl formamide in a conical flask. (0.21 g, 0.01 mol) of sodium azide and 0.5 g of zinc chloride were added. Then, 5 mL of carbon disulfide was added and mixed well. The mixture was kept at room temperature for 10–12 h. Then, the mixture was added in ice-cold water. The azide product was collected and used for further reaction. The compound was characterized by TLC, UV, IR, and melting point determination (Tables 3 and 4). Mobile phase of TLC was hexane:ethyl acetate (3:2) [4] Scheme 4.

### Preparation of 5-azido-1,3-benzoxazol-2(3 *H*)-one (P3B)

(1.69 g, 0.01 mol) of P1A was dissolved in 5 mL of dimethyl formamide in a conical flask. (0.21 g, 0.01 mol) of sodium azide and 0.5 g of zinc chloride were added in the above solution. Then, 5 mL of carbon disulfide was added and kept the mixture at room temperature for 10–12 h. Then, the mixture was added in ice-cold water. The azide product was collected and used for further reaction. The compound was characterized by TLC, UV, IR, and melting point determination (Tables 3 and 4). Mobile phase of TLC was hexane:ethyl acetate (3:2) [4].

### Preparation of 3-benzyl-5-(1 *H*-1,2,3-triazol-1-yl)-1,3-benzoxazol-2(3 *H*)-one (P4A)

(2.66 g, 0.01 mol) of P3A was dissolved in 10 mL of absolute ethanol and it was refluxed with (0.86 g, 0.01 mol) of vinyl acetate (density 0.93 g/cm^3^) for 8 h. The reaction was monitored by TLC. Mobile phase was hexane:ethyl acetate (2:1). Then, the product was collected from ice-cold water and recrystallized from rectified ethanol. The compound was characterized by TLC, UV, IR, MASS, NMR, and melting point determination [8].

### Preparation of 5-(1 *H*-1,2,3-triazol-1-yl)-1,3-benzoxazol-2(3 *H*)-one (P4B)

(1.76 g, 0.01 mol) of P3B was dissolved in 10 mL of absolute ethanol and it was refluxed with (0.86 g, 0.01 mol) of vinyl acetate (density 0.93 g/cm^3^) for 6 h. The reaction was monitored by TLC. Mobile phase was hexane:ethyl acetate (3.5:1.5). Then, the product was collected from ice-cold water and recrystallized from rectified ethanol. The compound was characterized by TLC, UV, IR, MASS, NMR, and melting point determination [8].

### Preparation of 4-amino-*N*-(3-benzyl-2-oxo-2,3-dihydro-1,3-benzoxazol-5-yl)benzenesulfonamide (P5A)

(2.59 g, 0.010 mol) of P2A was dissolved in 10 mL of absolute ethanol. Equal weight of potassium carbonate (anhydrous) and (1.72 g, 0.010 mol) of sulfanilamide was added and the whole mixture was refluxed for 3 h and the reaction was monitored by TLC. Mobile phase was hexane:ethyl acetate (3.5:1.5). Then, add the mixture in water and reprecipitate the product by acidifying with conc. HCl. Collect the crude P5A and recrystalize byrectified ethanol. The compound was characterized by TLC, UV, IR, MASS, and melting point determination [4,8].

### Preparation of 4-[(3-benzyl-2-oxo-2,3-dihydro-1,3-benzoxazol-5-yl)amino]benzoic acid (P6A)

(2.59 g, 0.010 mol) of P2A was dissolved in 10 mL of absolute ethanol. Equal weight of potassium carbonate (anhydrous) and (1.37 g, 0.010 mol) of *p*-aminobenzoic acid was added and the whole mixture was refluxed for 3 h and the reaction was monitored by TLC. Mobile phase was hexane:ethyl acetate (3.5:1.5). Then, add the mixture in water and reprecipitate the product by acidifying with conc. HCl. Collect the crude P6A and recrystalize by rectified ethanol. The compound was characterized by TLC, UV, IR, MASS, and melting point determination [4,8].

## Conclusions

Benzoxazole was reported as naturally occurring plant product and considered responsible for antibacterial activity in plant protection. We have synthesized and concluded its other synthetic derivatives. Compounds P4A and P4B have good antibacterial and antifungal activity, half of the Ampicillin and Cephalexin. P4A, P4B, P6A have good activity against *S. aureus* and *E. coli.* Compound P2B has good antifungal activity, half of the Miconazole against *C. albicans*. P2A, P2B, P5A, P6A have almost equal antibacterial activity.

## Competing interests

The authors declare that they have no competing interests.

## Authors' contributions

The CN Patel has guided this project. Both authors read and approved final manuscript.
